# Insights into the signal transduction pathways of mouse lung type II cells revealed by transcription factor profiling in the transcriptome

**DOI:** 10.5808/GI.2019.17.1.e8

**Published:** 2019-03-31

**Authors:** Chilakamarti V. Ramana

**Affiliations:** Department of Medicine, Dartmouth-Hitchcock Medical Center, Dartmouth Medical School, Lebanon, NH 03766, USA

**Keywords:** gene signature, lung injury, lung type II cells,microarrays, signal transduction pathways, transcription factor profiling

## Abstract

Alveolar type II cells constitute a small fraction of the total lung cell mass. However, they play an important role in many cellular processes including trans-differentiation into type I cells as well as repair of lung injury in response to toxic chemicals and respiratory pathogens. Transcription factors are the regulatory proteins dynamically modulating DNA structure and gene expression. Transcription factor profiling in microarray datasets revealed that several members of AP1, ATF, NF-kB, and C/EBP families involved in diverse responses were expressed in mouse lung type II cells. A transcriptional factor signature consisting of Cebpa, Srebf1, Stat3, Klf5, and Elf3 was identified in lung type II cells, Sox9+ pluripotent lung stem cells as well as in mouse lung development. Identification of the transcription factor profile in mouse lung type II cells will serve as a useful resource and facilitate the integrated analysis of signal transduction pathways and specific gene targets in a variety of physiological conditions.

## Introduction

The lung is a major organ involved in the critical function of respiration. In addition, the lung is also involved in removal of pathogens such as influenza and *Mycobacterium* as well as detoxification of environmental chemical pollutants accumulated in respiratory tissue [1-3]. Lung tissue contains airway and parenchyma compartments with more than 30 cell types including fibroblast, endothelial, epithelial, smooth muscle and macrophages [4-6]. Alveolar epithelium consists of two morphologically distinct type I and type II cells representing approximately 95% and 5% of the alveolar surface area, respectively [7]. Considerable progress has been made in understanding the role of type II cells in lung function and disease. Lung epithelium plays an important and active role in influenza virus and CD8+ T-cell mediated lung injury [8,9]. Surfactant is composed of phospholipids, proteins and carbohydrates and is mainly produced by lung type II cells. Surfactant promotes lung expansion, reduces edema and surface tension [10]. Lung type II cells participate in vesicular transport, lipid metabolism and detoxification. Furthermore, type II cells can undergo cell proliferation and transdifferentiate into type I cells in response to lung injury [11]. The development of type I and type II cell-selective monoclonal antibodies to apical surface membrane proteins such as T1-α and MMC4 facilitated investigating the role of lung epithelium in lung injury and repair [12,13]. Cell specific expression of a small number of genes such as surfactant proteins (SP-A, SP-B, and SP-C) in type II cells has been described [6,7]. Identification of sequence elements in the SP-C gene promoter and transcription factors that mediate tissue-specific expression in lung type II cells facilitated transgenic expression of heterologous genes [5,14]. Microarray technology allows for monitoring the transcriptional activity of several thousands of genes simultaneously [15]. Application of this technology to the lung developmental regulation, response to toxic chemicals and diseases have been described [16-18]. Furthermore, gene expression profile of human and mouse primary lung type II cells were reported [19-21]. Transcription factors are regulatory proteins that bind specific parts of the genome in tightly coiled structures called chromatin and regulate the availability of distinct stretches of DNA to be expressed in a tissue-specific manner. However, the role of transcription factors in the functional 2 organization of the mouse lung type II cell transcriptome is not well understood. The role of transcription factors such as Sox9 in pluripotent lung stem cells and AP1, activator transcription factor (ATF) in tissue-specific expression of Surfactant protein genes in type II cells has been demonstrated [22-24]. Isolation and characterization of lung stem cells capable of differentiating into alveolar epithelial type II cells has been described [22,25]. However, connecting the distinct profile of transcription factors and the gene expression within the transcriptome to elucidate biological functions of type II cells remains a major challenge. Gene knockout mice provided significant insights into the relative contribution of individual transcription factors into lung type II cell development and function [20,26,27]. Signal transduction pathways and transcriptional regulatory control of mouse lung type II cells were investigated using mouse lung and type II microarray data to gain novel insights into the biological organization of the transcriptome.

## Methods

### Animals and primary alveolar type II cell preparation

BALB/c mice (5–7 weeks old) were used. All experiments were conducted in strict accordance with the guidelines of the institutional animal care and use committee. Lung primary alveolar type II cells were prepared as described [7,21]. Briefly, lungs from BALB/c mice were dissected and put in a sterile culture tube containing serum-free Dulbecco’s modified Eagle’s medium (DMEM) and dispase and incubated for 45 min at room temperature. Lungs were then transferred to a culture dish containing DNAse1 (Sigma, St. Louis, MO, USA) and the tissue gently teased away from the airways. The cell suspension was successively filtered and then pelleted. Crude cell suspensions were added to culture dishes coated with anti-CD45 and anti-CD32 antibodies (BD Pharmingen, San Diego, CA, USA) and incubated for 1–2 h. Culture dishes were removed from the incubator, gently rocked to free settled type II cells and then resuspended in DMEM with 10% fetal bovine serum. Purity of the type II cell preparations 3 used for these studies was greater than 95% by morphological, immunocytochemical, and reverse transcriptase PCR assays of selected cell-specific markers criteria.

### Gene expression profiling

Total RNA was prepared from lung type II cells by using the Qiagen Rneasy kit (Valencia, CA, USA). For cDNA synthesis, RNA (10 μg) was annealed to the oligodT-T7 promoter at 70℃ for 10 min and then reverse transcribed at 42℃ for 3 h. The resulting double-stranded cDNA was used as a template to generate biotinylated cRNA from an in vitro transcription reaction using the Enzo Diagnostics RNA transcript labeling kit (Farmindale, NY, USA). The biotin-labeled cRNA were purified and hybridized to the murine U74A genomic array as directed by Affymetrix technical procedures manual (http://www.affymetrix.com). The washed arrays were stained with phycoerythrin-streptavidin and scanned by using an Affymetrix Gene-array scanner. The experiment was performed with RNA from lung type II cells pooled from 3 animals each for a chip and a total of 4 chips were used with similar results.

### Microarray data analysis

Scanned images were analyzed with Affymetrix microarray analysis suite 6.0 gene expression software. The gene list was selected based on the signal intensity of >700 arbitrary units and consistency of the signal intensity across the four chips. The signal intensity was normalized to glyceraldehyde-3-phosphate dehydrogenase (Gapdh) levels. Functional organization of the transcriptome was established using Database for Annotation, Visualization and Integrated Discovery (DAVID) bioinformatics [28]. Affymetrix identifiers (ID) of transcription factors were analyzed in the functional annotation tool of the DAVID 6.8 website (http://david.abcc.ncifcrf.gov). The software generated annotated chart with biological terms and p-value (Benjamini-Hochberg corrected for false discovery rate) associated with the transcriptional factors. Selected examples were shown. Expression profiling of a variety of mouse tissues has been described previously [29]. Tissue-specific gene expression dataset from multiple mouse tissues was downloaded from the website (http://www.biogps.org). Mouse lung type II data were normalized to 4 Gapdh levels and resulting data were analyzed by linkage across arrays and genes by using CLUSTER software [30].

Transcription factor interactions were visualized using protein-protein interactions databases (https://openwetware.org/wiki). Microarray datasets for mouse developmental stage E12.5 and adult CDH+, CDH–; EpCAM+, EpCAM– Sox9+ lung stem cells as well as in vitro differentiated E12.5 stage Sox9+ cells at passage 2 and 10 (P2, P10) were previously reported [22]. Mouse lung developmental gene expression dataset was downloaded from Jackson Lab website (http://www.jax.org) and analyzed using software from the website (http://www.heatmapper.ca).

## Results and Discussion

**Fig. 1.**
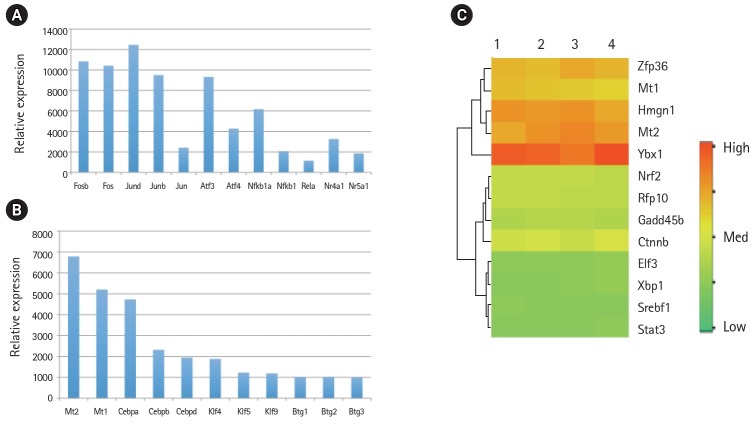
Relative mRNA expression levels of transcription factors in the mouse lung type II transcriptome. (A) The mRNA levels of activator protein (AP1), activation transcription factor (ATF), nuclear factor-κB (NF-κB), and nuclear hormone receptors (NR) family members involved in inflammatory response. (B) The mRNA levels of Mt, Cebp, Klf, and Btg family members of transcription factors. (C) High, medium, and low expression of transcription factors in four separate RNA preparations of mouse lung type II cells represented by red, yellow and green colors, respectively.

**Fig. 2.**
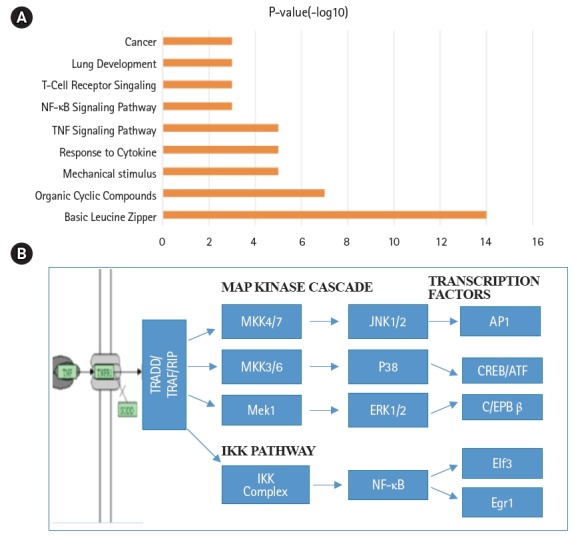
Annotation of biological functions and signal transduction pathways in mouse lung type II cells. (A) Signaling pathway terms associated with transcription factors of mouse lung type II were ranked by significance. (B) Schematic representation of tumor necrosis factor (TNF-α) mediated intracellular signal transduction pathways in mouse lung type II cells. NF-κB, nuclear factor-κB; MAP, mitogen-activated protein.

**Fig. 3.**
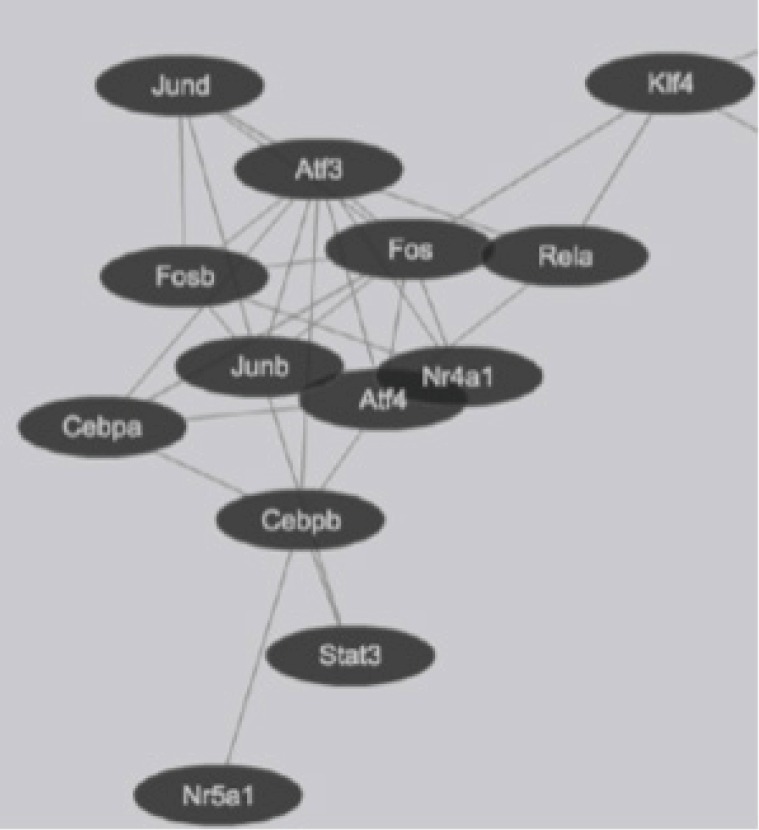
Transcription factor interactions in mouse lung type II cells predicted from Protein Interaction Databases. Transcription factors are represented by ovals and protein-protein interactions represented by connecting lines.

Global gene expression in mouse lung type II cells was examined using oligonucleotide microarrays in order to gain insights into the expression levels of transcription factors and signal transduction pathways critical for the transcriptome. Transcript analysis revealed that approximately 2,000 genes (16.6%) were expressed in type II cells, out of the 12,000 genes represented on the U74A mouse Affymetrix chip. About 575 genes were highly expressed whereas about 1,425 were expressed at low levels. Furthermore, a master list of about 350 known and highly expressed genes was constructed (Supplementary Table 1). This list includes about 50 transcription factors that were highly expressed in mouse lung type II transcriptome. Transcriptional factors bind to upstream regulatory elements such as promoters and enhancers and in co-operation with co-regulators and general transcriptional factors regulate gene expression [31]. The transcription factor profile of type II cells includes several members of the activator protein (AP1), ATF, nuclear factor-kappaB (NF-kB), Kruppel-like zinc-finger proteins (KLF), and CAAT/enhancer binding proteins (c/EBP) families. A general feature of the transcriptome is the differential expression of multiple members of a transcription factor family that controls redundant, overlapping or non-redundant functions (Fig. 1A and 1B). These include AP1 (Fos, Fosb, jun, Junb, and Jund), ATF (Atf3 and Atf4), NF-kB (Nfkb1 and Rela), and nuclear receptor or NR (Nr4a1 and Nr5a1) 5 family members. Many of these transcription factors have a prominent role in immune responses. Protein domain analysis revealed that the transcription factors have specific domains (leucine zipper, helix-loop-helix, zinc finger, Rel or Kruppel) that facilitate extensive homo-dimerization (ex., c-Jun) or hetero-dimerization (ex., Fos-Jun) that further expands the repertoire of gene regulation. Members of cellular enhancer binding protein or C/EBP family are involved in the regulation of multiple cell types [26]. Metallothioneins (Mt) are regulated by heavy metals and inflammatory response [32]. Nrf2 is a transcription factor with a basic-leucine zipper domain and regulates a large number antioxidant and xenobiotic-metabolizing enzyme genes through the antioxidant response element (ARE) [27]. Nuclear Receptor family members (Nr4a1 and Nr5a1) are highly expressed in lung epithelium [33]. KLF family members such as Klf4, Klf5 and Klf9 are implicated in the regulation of cell growth, differentiation and apoptosis [34]. Mammalian Btg/Tob family regulates transcription in the nucleus and messenger RNA deadenylation and implicated in anti-proliferation in multiple cell types [35]. Expression levels of transcription factors could vary significantly from high such as Y-box1 (Ybx1) and high mobility group (Hmgn1); moderate such as catennin β (Ctnnb) and metallothionein1 (Mt1) or low like Stat3 and sterol regulatory element binding protein (Srebf1) in type II cells (Fig. 1C). Cluster analysis of transcription factors with biological terms in DAVID Bioinformatics revealed that hetero-dimerization leucine zipper domain as a key regulatory property present in AP1, ATF, and NR families. Furthermore, protein phosphorylation, metal binding and dimerization are major regulatory mechanisms of this category (data not shown). Functional properties of type II cells such as detoxification (response to organic cyclic compounds), cytokine signaling (tumor necrosis factor or TNF-α) and cell growth (development and cancer) were also well represented based on p-value (Fig. 2A). Mapping the list of transcription factors on to Kyoto Encyclopedia of Genes and Genomes (KEGG) pathways revealed that TNF-α is the major signal transduction pathway functional in lung type II cells (Fig. 2B). TNF receptor 1 (TNFR1) is ubiquitous and is expressed on lung epithelial cells [9]. TNF signaling is mediated through TNFR1 to regulate mitogen-activated protein kinases including c-Jun N-terminal kinase (JNK), extracellular receptor kinases (ERK), p38 and IκB kinases to activate NF-kB [36,37]. In addition to NF-kB, TNF-α also activates AP1 via JNK and ATF via JNK, ERK, and p38 mitogen-activated protein (MAP) kinase pathways [36-38]. Involvement of ERK, JNK, p38 MAP kinases and transcription factors AP1, NF-kB, and ATF has been described in response to influenza infection [39]. TNF-α plays an important role in CD8+ T-cell mediated lung injury [21]. In a mouse transgenic model, lung injury was largely mediated by chemokines expressed by the epithelial cells upon CD8+ T-cell recognition and this response was dependent on TNFR1 [9]. Protein-protein interactions play an important role in co-operative and functional diversity of transcriptional networks in biological functions of multiple cell types [31]. Network analysis of transcription factor interactions in protein interaction databases revealed that extensive interactions between AP1, ATF, NF-kB, NR, and C/EBP family members are possible (Fig. 3). Furthermore, these interactions also predict a potential role for Atf3, Nr4a1, and AP1 family members in TNF-α induced CD8+ T-cell mediated lung injury.

**Fig. 4.**
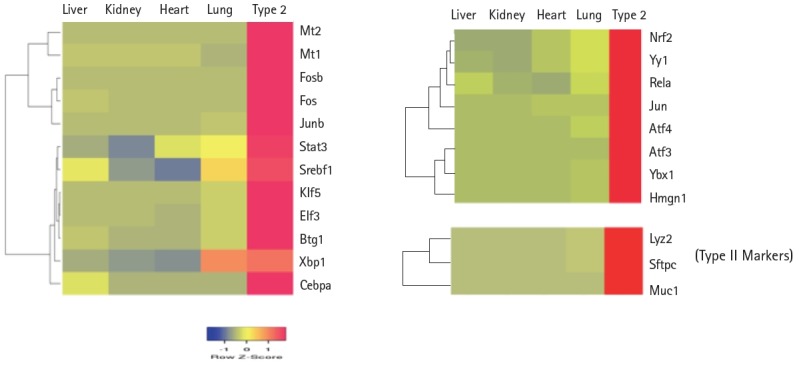
Tissue-specific expression of transcription factors. Cluster analysis of transcription factor levels in multiple mouse tissues. Lung type II markers such as surfactant protein C (Sftpc), mucin (Muc1), and lysozyme (Lyz2) were included for the purpose of comparison.

**Fig. 5.**
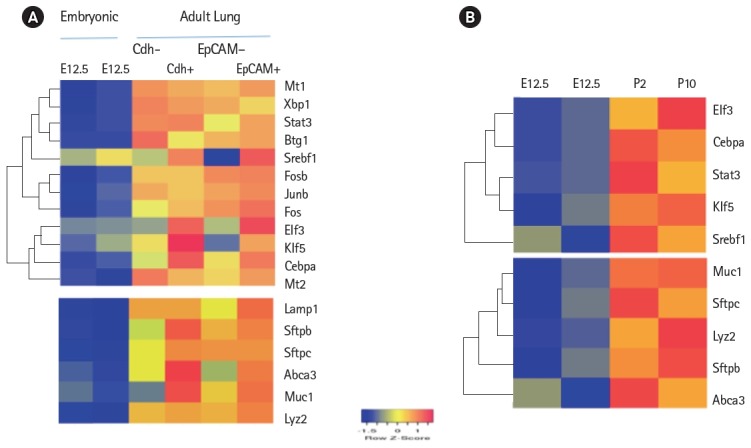
Heat Map representation of differentially expressed genes between E12.5 development and adult lung Sox9+ progenitors *in vivo*. (A) Cadherin (Cdh) and epithelial cell adhesion molecule (EpCAM) positive (+) and negative (–) adult epithelial cell populations were shown. (B) E12.5 Sox9+ progenitor cells after early (P2) or late (P10) cell passage in chemically defined medium in vitro. Lung type II gene markers such as Sftpc, Muc1, and Lyz2 were included for the purpose of comparison. Representative genes with differential expression are shown. Raw data values were normalized and are shown as rlog and a >2-fold change were represented by blue and red colors in either direction.

In gene expression analysis, clustering algorithm is often used to discriminate genes that are co-regulated in the experimental conditions studied [30]. Furthermore, cluster analysis in multiple tissues facilitates detection of overlapping and tissue-specific patterns of gene expression. This analysis revealed that the gene expression profile of lung and type II were closely related among all tissues tested (Supplementary Fig. 1). Consistent with these observations, shared transcription factor co-expression profiles include Xbp1, Stat3, and Srebf1. Furthermore, transcripts of Mt1, Atf3, Ybx1, and Hmgn1 levels were highly expressed in lung type II compared to the lung (Fig. 4). Cluster analysis also showed examples of highly expressed genes in type II cells compared to lung included SP-C, mucin1 (Muc1), lysozyme2 (Lyz2), ATP binding cassette transporter (ABCA3), sodium-phosphate co-transporter, hemolytic component, heat shock protein (84 kDa), catenin-src, Gro1, and Rab9 (Figs. 4 and 5, Supplementary Fig. 1). There is an overlapping gene expression profile signature between liver and type II cells (data not shown). Liver and lung type II have also a common role in lipid metabolism and express high level of transcription factors Cebpa and Srebf1 that are implicated in lipid metabolism [26,40].

**Fig. 6.**
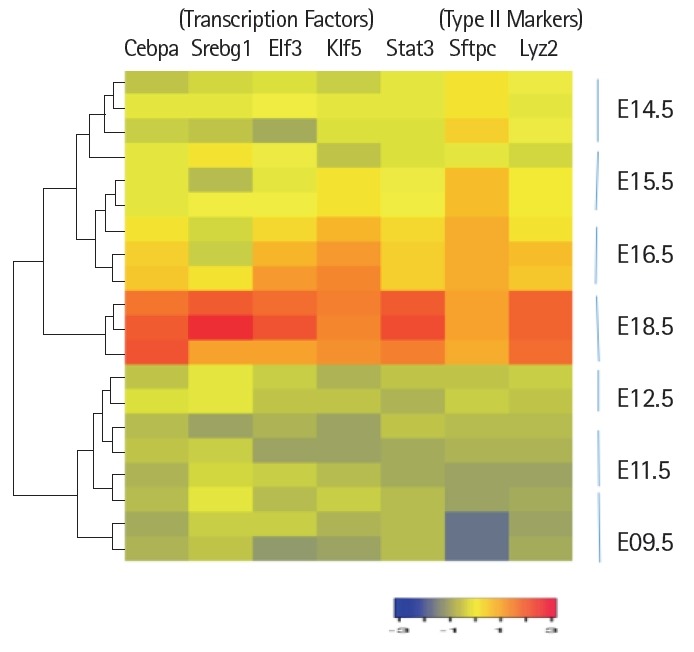
Heat map representation of differentially expressed transcription factors during mouse lung development. Raw data values were normalized and are shown as rlog and a >2-fold change were represented by blue and red colors in either direction. Transcription factors with differential expression are shown during development. Lung type II markers such as Sftpc and Lyz2 were included for the purpose of comparison.

**Fig. 7.**
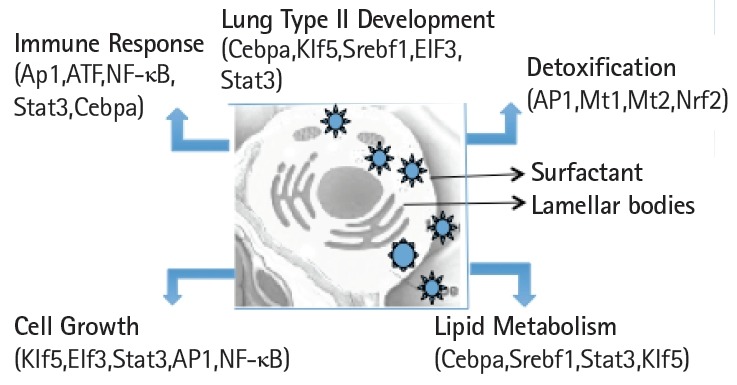
Schematic view of the transcription factors network in the functional organization of the transcriptome in mouse lung type II cells.

Expression levels of cell surface adhesion molecules such as E-cadherin (Cdh) and EpCAM are useful for staining and sorting lung epithelial cells [22]. A highly conserved cellular process in development known as epithelial-to-mesenchyme transition is responsible for transforming epithelial cells in metastatic lung cancer and tumor [41]. Sex determining region Y-box 9 (Sox9) was originally known for its functions in embryonic development specifically in bone formation, testis and lung development [42]. Sox9 is important for stem cell maintenance, tumor progression and metastasis [43]. Pluripotent Sox9 positive lung stem cells are capable of generating airway and type II cells in mice and transplantation of the Sox9+ human lung stem cells in cancer patients have been described [22,25]. I have investigated the transcription factors signature characteristic of lung type II cells in Sox9 positive stem cells of E12.5 lung mouse development and adult lung stem cells from mice [22]. Cluster analysis showed that the gene signature consisting of Stat3, Srebf1, Elf3, Klf5, Cebpa was highly expressed in adult mouse lung stem cells sorted for epithelial markers Cdh or EpCAM (Fig. 5A). Consistent with these results, lung type II marker genes such as surfactant protein C (sftpc), Mucin (Muc1), lysozyme 2 (Lyz2) were also highly expressed in adult compared with E12.5 Sox9+ stem cells. Under defined chemical conditions, E12.5 Sox9 positive lung stem cells can be differentiated into type II cells in vitro, after 2–10 passages in cell culture [22]. Interestingly, the same five transcription factors were also highly expressed under these conditions (Fig. 5B). A major disadvantage of many transcriptome data studies is that they provide a snapshot of gene expression profile in cells or tissues. However, it is well known that gene expression is dynamically and temporally regulated during mouse development. For example, gene expression profile of proteins encoding extra-cellular matrix varies significantly during lung development [16]. Klf5 is expressed at higher levels in lung epithelial cells at the E18.5 stage of mouse development and mice lacking Klf5 die at the time of birth due to respiratory distress [34,44]. Data mining of the transcription factor signature of Stat3, Srebf1, Elf3, Klf5, and Cebpa within the gene expression data set of mouse lung developmental stages revealed that the expression was relatively low in early developmental stages such as E9.5–E12.5, moderate in E14.5–E15.5 and relatively high during late embryonic developmental stages of E16.5–E18.5 (Fig. 6). These results suggest that the gene expression levels are dynamic over time in 8 different stages of mouse lung development. Transcription factor profile is intimately linked with the gene expression profile of any given cell type. Unique combination of transcription factors assembled on individual gene promoter determines the level of gene activity [23,31]. Furthermore, functional organization of mouse lung type II transcriptome can be elucidated in terms of potential role of specific combination of transcription factors. Multiple transcription factors have been shown to play an important role in the lung type II specific cell functions such as detoxification, lipid metabolism, immune responses and cell growth as revealed by knockout mouse models, as discussed below (Fig. 7).

### Detoxification

Proteins involved in detoxification and antioxidant defense are abundant in lung type II transcriptome. The redox system of glutathione (GSH) consists of primary and secondary antioxidants, including GSH peroxidase, GSH reductase and GSH S-transferase [45]. Depletion of GSH in the lung is associated with the increased risk of lung injury and disease [46]. Nrf2 plays a major role in detoxification by sequence-specific binding to ARE and up-regulating several genes [47,48]. Pathogenesis of lung diseases is tightly linked to exposure to environmental chemicals which require an enzymatic activation to exert their deleterious effects on pulmonary cells [49]. Nrf2 has a potential chemo-preventive activity, by upregulating antioxidant defenses and attenuating of inflammation and oxidative stress. Deficiency in Nrf2-GSH signaling attenuates type II cell growth and enhances sensitivity to oxidants implicated in cigarette smoke induced emphysema [46,48]. Metallothionein regulates the intensity of the induction of inflammatory proteins such as chemokines and cytokines. In Mt knockout mice the induction of cytokines and chemokines as well as pulmonary inflammation were greater in response to bacterial lipopolysaccharide. Mt protects against coagulation and fibrinolytic disturbance and acute lung injury by inhibition of pro-inflammatory mediators [50]. Knockdown of Nr4a1 in human lung epithelial cells resulted in significant increase in IkB-α phosphorylation and degradation resulting in enhanced NF-kB activity, whereas Nr4a1 overexpression decreased NF-kB activity [33,51]. Nr4a1 knockout mice show significantly enhanced allergic airway inflammation and aggravated mucus production [33].

### Lipid metabolism

Alveolar type II cells convert glycogen into phospholipids that are a major component of the surfactant proteins [8,52]. Keratinocyte growth factor (KGF) stimulates lipid metabolism in alveolar type II cells cultured on a matrix of collagen and matrigel [53]. Lung type II cells express lipogenic enzymes such as fatty acid synthase (Fasn) and stearoyl-CoA desaturases (Scd1, Scd2) and transcription factors such as C/eba, Klf5, Srebf1, and Stat3 that are implicated in fatty acid and phospholipid synthesis [20,26,40]. It is interesting to note that Cebpa, Fasn, and Scd1 are also highly expressed in brown fat, adipose and liver tissues that are involved in lipid and energy metabolism [54]. Cebpa knockout mice display abnormalities in liver and adipose tissues as well as hyper proliferation of lung type II cells and disturbed alveolar architecture resulting in perinatal death [26,55]. Klf5 is required for lung development and knockout mice die perinatally due to respiratory distress and also have defects in surfactant and lipid metabolism [44]. Stat3 regulates Abca3 transporter expression and influences lamellar body formation in alveolar type II cells [20,56].

### Immune responses

Alveolar type II cells secrete a variety of anti-inflammatory and antimicrobial substances into the alveolar fluid including surfactant proteins, lysozyme, lipocalin 2 and reduced GSH. A variety of histocompatibility antigens involved in antigen processing and adhesion molecules involved in inflammation were also expressed in type II cells. Pathogen response was mediated by chemokine-guided neutrophil and macrophage influx in pathogen response of lung type II cells [9]. AP1, ATF, and NF-kB transcription factors have a major role in influenza virus and cytokine induced signal transduction pathways and is consistent with the functional role of alveolar epithelium in virus and CD8+ T-cell mediated lung injury [8,21].

### Regulation of cell growth

Lung type II cells can undergo cell proliferation and differentiate into type I cells [11]. Several growth-associated genes regulate cell proliferation, growth arrest and apoptosis in the lung. These include KGF, hepatoma derived growth factor (Hdgf), heparin binding epidermal growth factor (Hb-egf), and amphiregulin (Areg). Previous studies have shown that keratinocyte and hepatocyte growth factors play an important role in lung development, inflammation and repair [53]. Klf5 is part of the pluripotent stem cell gene signature along with Oct4, Sox2 and, Myc that is required for perinatal lung morphogenesis and function [44]. AP1, NF-kB, ATF family members have been shown to participate in a variety of growth factor, stress response and cell death pathways [36,37]. Elf3 is a member of Ets family of transcription factors and is highly expressed in epithelial tissues. High level of Elf3 expression in mouse embryogenesis and 30% fetal lethality in homozygous knockout mice suggest important role in early development [57]. Furthermore, Elf3 regulates non-small cell lung carcinoma by modulating oncogenic signal transduction pathways [58].

Global gene expression profiling studies revealed that transcription factors regulate the functional organization of the transcriptome in mouse lung type II cells and the list provides an important resource. Taken together, transcription factor profiling in signal transduction pathways involved in lung injury as well as development expand our view of the transcription factor landscape in the regulatory genome architecture of mouse lung type II cells. A comprehensive transcriptome resource of many cell types in humans and mice called Cell Atlas is being developed [29,59]. This approach may be used for constructing novel signal transduction pathways from transcription factor profiles in developmental and ligand inducible gene expression microarray datasets. Further studies may provide a framework for generating novel mouse models for human lung diseases and potential therapeutic targets.

## Supplementary Materials

Supplementary Table 1. Functional organization of the transcriptome of mouse lung type II cells

Supplementary Fig. 1. Cluster analysis of gene expression in lung type II cells in comparison with other mouse tissues.
