# Supplementary material for: Insights into the signal transduction pathways of mouse lung type II cells revealed by transcription factor profiling in the transcriptome
Source: Genomics Inform. 2019 Mar 31;17(1):e8. doi: 10.5808/GI.2019.17.1.e8 (PMC6459171; doi:10.5808/GI.2019.17.1.e8)
Supplement: Supplementary Table 1. — Functional organization of the transcriptome of mouse lung type II cells [file gi-2019-17-1-e8-suppl1.pdf]

Supplementary Table 1. Functional organization of the transcriptome of mouse lung type II cells

Affymetrix ID	Signal Intensity	Signal Intensity	Signal Intensity	Signal Intensity	Description
Transcription					
102362_i	4133.8	5491.8	3802.6	4600.8	Jun-B oncogene
102364_	12629.8	14144.1	9871.9	13177.5	Jun proto-oncogene related gene d1
100130_	2594.9	2431.7	2576.6	2040.7	Jun oncogene
160901_	10202.4	10853.7	8848.8	11792.6	FBJ osteosarcoma oncogene
103990_	9823.3	12844.3	9670.8	11021.9	FBJ osteosarcoma oncogene B
101554_	6269.1	6923.5	5476	6037.6	I-kB alpha
98427_s_	858.8	849.2	1107.1	792.1	NF-kB,p105
97813_at	2192	1763.1	2497.2	1830.8	avian reticuloendotheliosis viral (v-rel) oncogene homolog
98447_at	4333.2	4925.2	5085.3	4605.5	C/EBP,alpha
92925_at	2219.2	2484.7	2486.3	2102.1	C/EBP,beta
160894_	2099.6	2246.1	1768.4	1681	C/EBP,delta
104155_f	11360.4	9533.5	7813.3	8603.8	activating transcription factor 3
100599_	4852.7	4056.6	4301.3	3880.5	activating transcription factor 4
160108_	3025.4	2895.3	2713.1	3316.1	p8 protein
101665_	1741.1	2160	1583.9	1965.2	nuclear receptor subfamily 5, group A, member 1
102371_	3347.4	3140.4	3338	3252.9	nuclear receptor subfamily 4, group A, member 1
93573_at	5774.4	4816.6	4827.9	5395.4	metallothionein 1
101561_	7884	6248.2	5409.1	7614.4	metallothionein 2
95682_at	1877.9	2415.7	1920.3	1874.1	damage specific DNA binding protein 1 (127 kDa)
161666_f	2626.3	2505.2	2128.6	2631	growth arrest and DNA-damage-inducible 45 beta
99622_at	1835.1	2358.8	1539.9	1809.3	Kruppel-like factor 4 (gut)
97937_at	1082.8	1253.6	1119	1457.8	Kruppel-like factor 5
93528_s_	870.9	1583	859.4	1448.7	Kruppel-like factor 9
93104_at	1012	942.3	1046	1045.5	B-cell translocation gene 1, anti-proliferative
101583_	3737.3	4795.8	3088.8	4777	B-cell translocation gene 2, anti-proliferative
96146_at	1030.7	965	1139.7	835.7	B-cell translocation gene 3
99452_at	948.3	1033.7	945.4	994.7	SREBP-1c
92697_at	616.4	684	767	631.5	forkhead box A1
93950_at	917.8	712.9	737.6	1055.1	forkhead box A2
98831_at	729.9	995.6	784.3	1031.1	forkhead box J1
160535_	438.6	861.7	568.3	813.9	nuclear factor, erythroid derived 2,-like 1
93740_at	7998.6	10921	7345.3	7633.7	Y box protein 1
94821_at	1206.7	963.7	1337.2	864	X-box binding protein 1
95124_i_	901.2	1146.3	895.8	1009.3	ring-box 1
100553_	1098.8	1045.1	914.8	1024.6	ret finger protein
92830_s_	6807.9	6506.8	4974	5008.7	zinc finger protein 36
96859_at	2941.4	3154.6	2604.1	2754.9	ring finger protein 10
93547_at	1807.5	1755.2	1570.3	1804.1	core binding factor beta
160430_	3284.2	2950.7	4571.1	3309.1	catenin beta
100554_	1317.8	898	1254.9	852.9	PDZ and LIM domain 1 (elfin)
99099_at	888.8	1112.4	863	921.5	signal transducer and activator of transcription 3

99059_at	1098.1	1482.9	937.5	1126.8	E74-like factor 3
98767_at	391.9	468.5	587.4	534.9	YY1 transcription factor
99532_at	1711	2357.8	2185.7	1988.7	transducer of ErbB-2.1
101086_f	1196.1	1652.9	1640.4	1422.6	cellular nucleic acid binding protein
101973_	1155.4	1103	1359.1	1046	CBP/p300
104331_	755.8	1302.8	943.3	1167.7	SWI/SNF related
96699_at	7169.3	5842.3	7495.8	6107.5	high mobility group nucleosomal binding domain 1
101634_	1598.7	1142	1684	1346.	nucleophosmin 1
Detoxification					
102847_	934	1619.3	1212.9	968	cytochrome P450, 2a4
102701_	3808.2	3747.9	2542.1	2327.6	cytochrome P450, 2b10, phenobarbitol inducible, type b
100069_	9020.1	8391.8	8118.5	12234.9	cytochrome P450, 2f2
103353_f	5227.8	3648.5	3574.2	3611.7	P450, subfamily IV B, polypeptide 1
99583_at	3439.4	2812	3196	2866.4	glutathione S-transferase, pi 1
93543_f_	9270	9289.9	7351.1	12457.1	glutathione S-transferase, mu 1
93009_at	1426.9	1066	1306.5	1339.5	glutathione S-transferase, mu 2
97681_f_	878.5	640.7	784	1038.2	glutathione S-transferase, mu 3
160350_	1444.3	1359.8	1486.1	1154.8	glutathione transferase zeta 1 (maleylacetoacetate isomera
95019_at	940.8	1010.6	1076.6	779.7	glutathione S-transferase, theta 1
97819_at	1547.2	1661.9	1735.9	2717.9	glutathione S-transferase like
101676_	3310	2789.6	5145.8	2798.4	glutathione peroxidase 3
94897_at	1044.1	921.3	1038.9	792.9	glutathione peroxidase 4
96888_at	4325.2	4192.5	4475.2	4499.9	aldo-keto reductase family 1, member A4 (aldehyde reduct
100068_	11582	10855.4	10035.5	16551.9	aldehyde dehydrogenase family 1, subfamily A1
97055_s_	7262.8	7009.2	7174.3	7421.1	peroxiredoxin 1
100332_	2245.4	1205.3	1619.2	1178.8	peroxiredoxin 5, related sequence 3
95722_at	1104.3	1100.1	1032.7	703.6	glutaredoxin 2 (thioltransferase)
100538_	2684.9	2162.5	1638.8	2330.3	superoxide dismutase 1, soluble
160479_	1020.8	764.8	1391.3	1098.6	catalase 1
160101_	2085.5	1473.6	1869.8	1592.7	heme oxygenase (decycling) 1
101984_	1057.8	987	1310.5	769	ATX1 (antioxidant protein 1) homolog 1 (yeast)
92807_at	6418	5876.9	5784.4	6616.5	thioredoxin
161284_r	974.6	975.5	659.5	1034.3	thioredoxin reductase 1
100596_	5673.4	4931.8	4859.6	4417.1	selenium binding protein 1
160275_	1242.5	1099.7	1209.1	922.8	selenoprotein R
96895_at	1848.4	565.1	1992.2	1516.1	paraoxonase 1
94900_at	34063	43252.8	26450.3	47406.5	surfactant associated protein C
96092_at	12914	13576.5	11984.3	14787.2	haptoglobin
92851_at	798.3	1029.6	1036.9	1420.9	ceruloplasmin
99872_s_	12266.4	9370.2	9972.6	11619.8	ferritin light chain 1
94794_at	7131.3	6112.2	6919.8	6482.6	ferritin heavy chain
162457_	11896	11226	8444.4	9351.6	Hemoglobin alpha,adult chain 1
103534_	14226	11717	11292	11491	Hemoglobin beta, adult major chain
103958_	1503.9	1391.5	1364.3	1612.1	transferrin receptor
Membrane Protein					

161510_f	1164.7	1547.5	1058.3	1321.7	sodium channel, voltage-gated, type I, beta polypeptide
93798_at	3185.8	3529.8	4086.7	2959.1	ATPase, Na+/K+ transporting, alpha 1 polypeptide
99579_at	2441.8	2100	2370.8	2113.2	ATPase, Na+/K+ transporting, beta 3 polypeptide
103935_	974.1	1065.2	1330	1365.7	ATPase, Ca++ transporting, ubiquitous
102335_	1694.4	821.8	1127.6	870.2	potassium channel, subfamily K, member 1
94757_at	325.9	705.6	485.1	472.1	cystic fibrosis transmembrane conductance regulator
96919_at	6071.1	5247.4	5165.2	4538.3	ATPase, H+ transporting, lysosomal,16kD
95746_at	1637.3	2012.9	2438.4	1981.8	ATPase, H+transporting,lysosomal,70kD, isoform 2
92598_at	2605.9	2745.8	2928.2	2920.6	ATPase, H+transporting,lysosomal beta56/58 kD
160612_	746.9	1016.1	722.7	904.3	ATP-binding cassette, sub-family G (WHITE), member 1
93045_at	4634.5	5122	5147.6	4140.5	ATP-binding cassette, sub-family D (ALD), member 3
99500_at	1787.5	2644.9	2480.7	2757.6	solute carrier family 12, member 2
103918_	494	464.6	497.6	438.9	solute carrier family 15 (H+/peptide transporter), member 2
101877_	1776.6	1940.1	1862.4	1349.8	solute carrier family 31, member 1
98994_at	13333.9	15143.3	13030.6	15856.1	solute carrier family 34 (sodium phosphate), member 2
95473_s_	911.1	1189.4	1124.5	1141.5	FXYD domain-containing ion transport regulator 6
102804_	1365.9	1248.9	1453.9	1247.1	CEA-related cell adhesion molecule 1
103816_	897	942.6	652	1085.1	junction cell adhesion molecule1
102918_	10365.1	12540.7	10249.8	12277.4	mucin 1, transmembrane
94266_at	1955.4	1775.5	1621.1	1622.2	type I transmembrane protein Fn14
94493_at	4028.3	3538.6	3693	3338.1	claudin3
101410_	1950.7	1855.1	2630.2	2853.3	claudin4
99561_f_	1006.9	897.3	791.5	907.6	claudin7
101123_	4360.2	3179.1	3900.5	3320.1	integral membrane protein 2 B
100345_f	7082.7	6501.1	6287.9	5378.6	vesicle-associated membrane protein 8
94889_at	4071.7	2498.3	3752.3	3014.1	vesicle-associated membrane protein,33 kDa
96935_at	1433.9	1221.7	1452.3	1288.2	membrane-associated protein 17
97426_at	1571.3	1527.5	1711.4	1843.9	epithelial membrane protein 1
100064_f	2087.9	1950.2	2146.4	1463.6	gap junction membrane channel protein alpha 1
96283_at	1700.4	2063.1	2208.9	1763.7	integral membrane protein 3
161359_	3059.9	2870.6	2905.6	2643.1	podocalyxin-like
Immune response					
92866_at	10168.7	9877.5	7802.8	7593.7	histocompatibility 2, class II antigen A, alpha
100998_	2998.2	2612.5	2844.5	1823.4	histocompatibility 2, class II antigen A, beta 1
94285_at	3887.2	2948.1	3074.3	1841.1	histocompatibility 2, class II antigen E beta
97540_f_	9764.8	9569	8269.9	7025.4	histocompatibility 2, D region locus 1
93092_at	2368.1	1723.8	1852.7	1549.8	histocompatibility 2, class II, locus DMa
93120_f_	8928.5	7997.3	7829.4	6048.8	histocompatibility 2, K region
98035_g	1559.8	1576.7	1927.6	1015	histocompatibility 2, class II, locus Mb1
102161_f	2254.2	1565.9	1834.8	1068.4	histocompatibility 2, Q region locus 2
93078_at	5958.4	5382	4595.8	2232.8	lymphocyte antigen 6 complex
93077_s_	7038.3	5876	5134.9	3291.6	lymphocyte antigen 6 complex, locus C
101487_f	19526.6	17911.9	15353.7	18582.4	lymphocyte antigen 6 complex, locus E
101054_	13948.2	14806.3	11259	12424.3	Ia-associated invariant chain
93088_at	3138.4	2540.2	3628.7	2100.2	beta-2 microglobulin

https://doi.org/10.5808/GI.2019.17.1.e8

94206_at	2250.7	1910.9	2589.6	1967.5	B-cell receptor-associated protein 37
160257_	1367.2	1181.9	1269	1095.4	FK506 binding protein 1a (12 kDa)
99546_at	1421.5	1177.3	1443.4	1015.6	FK506 binding protein 2 (13 kDa)
92808_f_	2132.5	2679.8	2247.8	2400.5	FK506 binding protein 4 (59 kDa)
100613_	1421.8	1451.1	1389.9	1152.5	FK506 binding protein 8 (38 kDa)
103458_	4837.7	5569.3	6536.7	5037.1	hemolytic complement
97689_at	991.3	960.7	1354.5	1159.8	coagulation factor III
93497_at	774.9	1534.3	1070.5	1527.3	complement component 3
99475_at	2222.3	1482.2	2358.4	1512	cytokine inducible SH2-containing protein 2
99491_at	1206.1	1051.5	952.2	883	interleukin 10 receptor, beta
92858_at	1242.9	771	1357.3	996.7	secretory leukocyte protease inhibitor
100758_	15047.6	13580.4	11547.8	14249.6	Fas death domain-associated protein
95348_at	3087.9	1083.2	2275	1904.3	GRO1 oncogene
101560_	1589.6	1454.9	1427.6	1652.2	embigin
101078_	3385.7	3009.5	3700.9	3446.7	basigin
160463_	1806.9	1642.7	1643.4	1653.1	myeloid differentiation primary response gene 116
93604_f_	1529.1	2956.5	1970.2	2990.1	immunosuperfamily protein BI2
100540_	1173.4	920	1226.8	890.9	leukotriene A4 hydrolase
Ras/Rab family					
101030_	2195.9	2869.8	2785.6	2371	aplysia ras-related homolog B (RhoB)
101113_	1795.4	1993.6	1347.5	1054.4	aplysia ras-related homolog A2
101555_	2955.3	3213.7	3431.5	2582.8	RAS-related C3 botulinum substrate 1
102821_	1037.3	1142.1	1298.5	1298.7	RAS-like, family 2, locus 9
101254_	3370.7	3038.7	3333.6	3039.8	RAN, member RAS oncogene family
161304_r	869.7	1092.3	866.9	1009.7	guanine nucleotide binding protein, related sequence 1
94899_at	1686.3	1838.9	2583.3	1436.5	Rho interacting protein 3
94269_at	5413.5	5228	6201.6	4467.1	Rab acceptor 1 (prenylated)
94319_at	1194.2	1133.8	1277.1	1248.2	RAB18, member RAS oncogene family
94814_at	1658.9	1621.6	1740.4	1460.9	guanine nucleotide binding protein, alpha inhibiting 3
95516_at	1569.4	1294.5	1651.3	1039.7	RAB9, member RAS oncogene family
95442_at	2166.5	2120.1	2225.4	2144.3	RAB3D, member RAS oncogene family
96262_at	1102.6	1008.7	1288.9	873.9	RAB5C, member RAS oncogene family
99596_f_	3072.8	4316.6	4501.6	4164.2	guanine nucleotide binding protein, alpha inhibiting 2
99340_at	7857.6	5184.2	6811.1	6063.2	guanine nucleotide binding protein,beta2,related
96911_at	2569.7	2037.4	2097.7	1811.9	guanine nucleotide binding protein, beta 2
162468_	1368.3	1165.4	1034.6	1575.2	aplysia ras-related homolog 9 (RhoC)
100561_	935.5	1885.9	1542	1692.3	IQ motif containing GTPase activating protein 1
101016_	7080.2	6273.8	6819.6	6640.5	ADP-ribosylation factor 1
160082_	1045.9	1018.3	1343.2	969.9	ADP-ribosylation factor 4
160371_	3617.9	4221.5	2610	3297.9	ADP-ribosylation-like factor 6 interacting protein
92968_at	1842.1	1684.6	1573.9	1596.5	ADP-ribosylation factor 5
Calcium regulation					
96522_at	4773.8	3931.9	4916.9	4142.6	calmodulin
93293_at	10691.6	10054.5	9785.2	10216.2	calmodulin2
161703_f	1490.2	1817.1	2590.6	1758.3	annexin A1

100569_	6133.4	5614.1	6302.5	5183.7	annexin A2
101393_	1183.6	817.9	1374.3	744.9	annexin A3
100584_	2168.1	1741.2	2359.8	1574.6	annexin A4
93083_at	2192.2	2027.2	2128.5	2039.4	annexin A5
94304_at	1088.6	1123.6	1171.9	990.8	annexin A6
102815_	715.1	1011.6	1174.6	745.2	annexin A11
95453_f_	1057.3	980.5	1143	1040.5	S100 calcium binding protein A1
92770_at	2946.3	3083.3	2820.2	2916.1	calcium binding protein A6 (calcyclin)
98600_at	7684.4	7551.5	6596.2	7500.4	S100 calcium binding protein A11
100959_	1771.4	1920.9	1642.5	2014.5	S100 calcium-binding protein A13
Cytoskeletal					
92550_at	2837.4	2354.3	2703.5	2600.1	keratin complex 1, acidic, gene 19
160532_	1467.9	1605.8	1603.1	1757.5	tropomyosin 1, alpha
101093_	1117.9	1925	1247.4	2087.5	procollagen, type IV, alpha 1
161156_r	1458.5	1221.3	1116.6	1218.9	procollagen, type I, alpha 2
94492_at	7045.6	7413.3	7021.3	8182.8	destrin
94270_at	3205.5	2601.7	3348.8	3288.7	keratin complex 1, acidic, gene 18
95493_at	2037.1	1654.2	2073.6	1751.6	procollagen, type VI, alpha 1
96298_f_	7491.9	6135.4	6099.8	6430.8	dynein, cytoplasmic, light chain 1
99119_at	3287.6	2855.3	2829.6	2869.3	cofilin 1, non-muscle
95434_at	1544.9	1208.6	1221.3	1334.7	actin related protein 2/3 complex, subunit 3 (21 kDa)
92759_at	1080.8	971.6	1622.7	1126.7	laminin, beta 3
96016_at	1646.1	1351.1	1496.8	1283.8	laminin receptor 1 (67kD, ribosomal protein SA)
95286_at	1138.5	1342	1361.6	1447.7	clusterin
97904_at	1927.9	2153.6	2446.6	1975.2	actin-related protein 3 homolog (yeast)
93750_at	2303.6	2075.6	2590.8	2203.4	gelsolin
101039_	1119.2	1374.7	1400.7	1457.6	procollagen, type IV, alpha 2
101009_	2905.4	3076.5	3026.2	3081.4	keratin complex 2, basic, gene 8
98152_at	3359.2	2970.1	2768.4	2311.6	cateninsrc
98476_at	1054.8	1067.1	897.1	982.4	ankyrin 3, epithelial
100084_	1409.3	1841.5	1631	1852.3	villin 2
98988_at	1375.5	1023.3	1194	849	molecule possessing ankyrin-repeats
99013_f_	3258.1	3773.8	3486.7	3093	tropomodulin 3
160162_	4364.3	3598.7	3536.6	3350.8	transgelin 2
Protease					
102373_	3105.7	2300	3630.4	2104.2	glutamyl aminopeptidase
101019_	1592.8	1431.8	2119.8	1531.6	cathepsin C
100610_	1408.5	1336	1425.2	1150.8	calpain 4
101972_	20408.8	21224.2	17349.9	21446.4	kidney-derived aspartic protease-like protein
98405_at	1473.1	1228.2	1411.1	741.7	serine protease inhibitor 6
99993_at	1705.7	1647.4	1538.1	1472.7	alanyl (membrane) aminopeptidase
99586_at	3154.8	3074	3557.2	2699.3	cystatin C
93810_at	1934.1	2230	2062.5	1907.5	cathepsin D
94834_at	6461.7	4453.3	5062.9	3437.5	cathepsin H
100581_	3140.2	2455.6	3098.6	2582.1	cystatin B

Ubiquitin-proteosome					
101255_	23698.6	28217.3	19531.9	24260.8	ubiquitin B
95215_f_	12017.9	13938.2	11644.4	13521	ubiquitin C
101091_	1593.1	1137.8	1701.4	1468	ubiquitin-like 1
94018_at	1222.9	1150.4	1520.8	1147.4	ubiquitin-like 3
97479_at	1176.7	1318.2	1197.2	1107.3	ubiquitin-conjugating enzyme 7
101510_	1320.3	1647.5	1396.9	1363.7	protease (prosome, macropain) 28 subunit, alpha
100733_	1895.7	1578.4	2103.9	1494.4	proteasome (prosome, macropain) subunit, alpha type 2
92544_f_	1499.6	2213	1985.9	2596.9	proteasome (prosome, macropain) subunit, alpha type 3
96952_at	1330.4	942.8	1387.1	893.2	proteasome (prosome, macropain) subunit, alpha type 6
94025_at	1250.9	1069.8	1364	904.9	proteasome (prosome, macropain) subunit, beta type 3
98557_f_	1706.2	1465	1493.3	978.5	proteasome (prosome, macropain) subunit, beta type 4
101995_	3754.1	3287.3	3560.6	3353.1	sequestosome 1
93025_at	1457.2	1251.8	1539.3	1106.1	Nedd4 WW-binding protein 5
92863_at	2048.7	1602	1418.1	1994.4	WW domain binding protein 2
92619_at	1653.5	1651.3	1375.1	1482.3	WW domain binding protein 1
100522_	3229.2	3635.5	3074	3966.7	WW domain binding protein 5
Cell growth					
92629_f_	2678.9	3254.8	2710.9	3391.8	hepatoma-derived growth factor
92730_at	994.2	1671.5	1127.1	1551.3	heparin binding epidermal growth factor-like growth factor
92756_r_	1467.8	1282.5	854.5	1221.8	secretin
93091_s_	447.7	478.4	1499.3	425.6	fibroblast growth factor receptor 2
100123_f	3723.3	4767.3	4454.6	4950	integrin beta 1 (fibronectin receptor beta)
99915_at	843.9	1199.4	1017.8	1034.7	amphiregulin
98083_at	4326.4	5693.3	4469.4	5787.2	immediate early response, erythropoietin 1
99109_at	3881.3	3263.3	3598.1	3382.3	immediate early response 2
94384_at	11126.8	5914	7368.8	8537.7	immediate early response 3
92773_at	666.2	666.3	984.2	590.3	immediate early response 5
103904_	2493.1	2205	2250.1	2196.9	insulin-like growth factor binding protein 6
100566_	899.1	1611.3	1286.2	1957.9	insulin-like growth factor binding protein 5
100718_	4801.5	5043.7	4360.6	6087.6	prothymosin alpha
98129_at	1629.5	2120.4	1808.4	1916.1	thymosin, beta 10
98531_g	1558.8	1386.5	1748.1	1477.5	growth arrest specific 5
99067_at	1647.4	1685.4	1629.9	1654.5	growth arrest specific 6
96632_at	1852.7	2159.7	2265.1	2026	MORF-related gene X
93288_at	2006.1	2242.1	2119.3	2235.1	Bcl2-interacting killer-like
100151_	1815.1	1569.9	1888.2	1594.2	tumor differentially expressed 1
92625_at	1596.8	1190.8	1905.7	1157.2	NM23B,nucleoside diphosphate kinase
160499_	1491.4	1940.6	1926.9	2153.4	tumor rejection antigen gp96
98064_at	1140.4	1063.5	1113.8	1165.6	angio-associated migratory protein, related sequence
Lipid metabolism					
101044_	2705.2	1737.1	2698.4	2174.1	aminolevulinate, delta-, dehydratase
160832_	1422.1	1426.4	1509.4	1236.5	low density lipoprotein receptor
100893_	1064.3	922.4	1343.6	888.2	serine palmitoyltransferase, long chain base subunit 2
100927_	1776.8	1222.7	1521.5	1425.9	phospholipid transfer protein

93354_at	983.6	806.6	1133.9	735	apolipoprotein CI
93500_at	6998.8	7158.9	5989.2	7098.7	aminolevulinic acid synthase 1
93323_at	5949.1	4587.5	4967.7	4840.4	proteolipid protein 2
95758_at	4064.3	2940.8	3868.7	3939.8	stearoyl-Coenzyme A desaturase 2
95356_at	4128.4	3994.3	4636.2	3668	apolipoprotein E
97456_at	2164.7	1798.1	2895.2	1835.1	fatty acid Coenzyme A ligase, long chain 5
98575_at	1667.8	2999.2	2864.7	2069	fatty acid synthase
96735_at	1346.8	1181.1	1220.7	1160.8	phosphatidylcholine transfer protein-like
93754_at	1168.9	870.9	1412.4	865.9	enoyl coenzyme A hydratase 1, peroxisomal
162077_f	1163.4	1260.8	1254.3	1424.2	stearoyl-Coenzyme A desaturase 2
160564_	2320.2	2151.4	3317.2	1913	lipocalin 2
160120_i	2691.8	2375.2	2636.6	2314.4	phospholipase A2, group IB, pancreas
92768_s_	6328.8	3485.5	3574.5	2599.9	aminolevulinic acid synthase 2, erythroid
93278_at	929.6	1006.8	1709.3	932.6	sterol carrier protein 2, liver
98429_at	2073.9	1699.8	1496.9	1783.1	lysophospholipase 2
97114_at	1723.6	1803.9	1897.8	2387.1	prosaposin
Carbohydrate metabolism					
99952_at	7962.4	7273.9	8021.2	8477.5	chitinase 3-like 1
160509_	3119.7	1529.8	3045.1	1792.5	chitinase, acidic
101753_	29611.6	28502.8	23870.9	34682.4	P lysozyme structural
Amino acid metabolism					
101013_	9230.8	6971.8	8042.7	7701.9	ornithine decarboxylase antizyme
101002_	702	906.6	1445.7	840.4	ornithine decarboxylase antizyme inhibitor
92848_at	1840.2	1833.4	1879.7	1687.3	ornithine aminotransferase
94454_at	1732.3	1544.4	1790.1	1228.1	proline rich protein expressed in brain
101207_	11588	11693.9	11404.2	12195.7	peptidylprolyl isomerase A
100089_	1078.9	955.5	1152.1	1127.3	peptidylprolyl isomerase C
94915_at	1663.8	1419.6	1940.5	1520.9	peptidylprolyl isomerase B
100446_r	14487.6	11845	8298.2	9845.4	small proline-rich protein 1B
92608_at	2115.6	2053.5	2184.3	1903.2	cysteine rich protein
94061_at	2810.6	2047.2	2488.9	2586.4	cysteine rich intestinal protein
RNA metabolism					
94552_at	1516.5	1450.3	1846.2	1344.4	poly(rC)-binding protein 1
92621_at	2119.6	2209.2	2330.1	2188.8	poly(rC) binding protein 2
96041_at	1431.1	1247.5	1788	1246.4	RNA binding motif protein 3
96038_at	5701.5	4168.7	5975.8	4055	ribonuclease, RNase A family 4
Stress response					
95057_at	2311.6	1913.1	2670.2	1590.5	ER stress inducible, homocysteine-inducible
95359_at	7647.1	8051	6324.2	7167.8	ER stress inducible, homocysteine-inducible
93875_at	1364.5	1608	1241.1	1637.1	heat shock protein, 70 kDa 3
93324_at	1616.3	1457.6	1877.8	1585.8	butyrate response factor 1
93277_at	662.1	1430.4	1184.1	1022.5	heat shock protein, 60 kDa
93264_at	1623.9	1660.8	2110.9	1637.6	ethanol induced 6
160547_	1499.9	1879.9	1011.3	1343.6	upregulated by 1,25-dihydroxyvitamin D-3
160549_	1122.5	1172.4	1507.9	1329	TPA regulated locus

160383_	1204.9	1171.2	1109.3	987.9	silica-induced gene 81
104701_	872.9	1032.6	1153.1	940	stimulated by retinoic acid 14
101955_	1839.9	1991.7	2284.5	2512.4	heat shock 70 kD protein 5
102599_		18394.7	16448.5	21858.5	translationally regulated transcript (21 kDa)
Kinase regulation					
92310_at	1942.5	1406.9	1907.4	1708.6	serum-inducible kinase
97890_at	1206.5	1229.1	1366.1	1194.3	serum/glucocorticoid regulated kinase
101007_	1099.2	1242.6	1098.9	908.8	MAP kinase-interacting serine/threonine kinase 2
101448_	1753.1	2045.9	1666.5	2245.6	HGF-regulated tyrosine kinase substrate
104598_	4521.2	3669	3747.9	3800.5	protein tyrosine phosphatase, non-receptor type 16
92573_at	1947.9	1689.1	1581.7	1678.4	protein phosphatase 2,regulatory subunit A
93274_at	1527.5	1869	1846.4	1603.3	CDC-like kinase
93314_g	1734.6	1596.1	1601.6	1829.1	mitogen activated protein kinase kinase 3
95508_at	854.1	1069.6	1145.8	1107.4	NCK-associated protein 1
95721_at	1486.7	1184.2	1540.1	1388.4	MAP kinase-activated protein kinase 2
99458_i_	1069.6	1664.4	1429.6	1671.1	ELKL motif kinase
96852_at	1399.8	1442.5	1645.9	1292.4	cAMP dependent protein kinase
94489_at	2820.4	3162.8	3289.6	2953.4	protein tyrosine phosphatase 4a
organelle					
94876_f_	1033.1	1376.6	1411.2	1319.7	golgi reassembly stacking protein 2
99143_at	1921.5	2287	2672.7	1562.9	trans-golgi network protein 2
97444_at	985.9	1011.7	1384.5	975.9	lysosomal thiol reductase IP30 precursor
160089_	7809.3	7812.5	7507.8	8510	lysosomal membrane glycoprotein 1
96876_at	6327.4	6269.2	6227.5	5885.1	lysosomal-associated protein transmembrane 4A
93305_f_	2266	2270.1	2721.6	2034.3	vesicle-associated membrane protein 8
101590_	1433	1261.5	1849.5	1181.9	lysosomal membrane glycoprotein 2
160085_	1348.5	1358.6	1124.7	1283.3	thiosulfate sulfurtransferase, mitochondrial
100099_	2606.2	2104.6	2337.2	2117.4	sphingomyelin phosphodiesterase 1, acid lysosomal
100029_	1442.5	1319.7	1033.5	1414.9	peroxisomal biogenesis factor 14
101587_	1233.6	996.2	1494.7	1414.1	epoxide hydrolase 1, microsomal
94077_f_	1508.4	1968.8	1973.3	2541.5	ribophorin II
160552_	1014.7	1372.3	965.7	1133.1	adaptor protein complex AP-1, sigma 1
93362_at	2054.2	1792.3	1908	1706.2	adaptor protein complex AP-2, mu1
96019_at	1173.6	1182.7	1191.2	1061.5	pantophysin
93341_r_	2923.6	2124.3	2350.7	2780.8	coatomer protein complex, subunit beta 2 (beta prime)
97882_at	1930.3	1915.6	1916.9	1815.5	SEC61, alpha subunit (S. cerevisiae)
93295_at	976.1	645.1	1332.8	694.6	chaperonin subunit 5 (epsilon)
100579_	1123.3	1034	934.5	1052.2	clathrin, light polypeptide (Lca)
104514_	1956.5	1976.4	1699.7	1822.7	epsin 1
160543_	3333.9	2624.2	2942.7	2514.6	sorting nexin 3
Unassigned					
101585_	1068	948.5	1169.7	1386.3	progesterone receptor membrane component
104680_	2252.1	1355.7	2130.3	1694.8	receptor (calcitonin) activity modifying protein 1
160194_	1694.5	2199.5	1345.8	1870.1	glutaryl-Coenzyme A dehydrogenase
93728_at	2888.1	3015.2	2902.3	3248.5	transforming growth factor beta 1 induced transcript 4

95014_at	911.2	993	1373.6	1189.7	f-box only protein 6b
96012_f_	1719.2	2757.2	2348.2	2636.9	matrin 3
96657_at	4612.7	4684.3	3778.9	3503.3	spermidine/spermine N1-acetyl transferase
96869_at	3390.8	2900	3152.2	2968.8	gamma-aminobutyric acid receptor associated protein
97544_at	3784	3891.3	2347.1	3543.2	tyrosine 3-monooxygenase activationprotein,zeta
99581_at	3829.9	2677.1	4150.4	2580.1	histidine triad nucleotide-binding protein
100710_	850.1	1071.5	1018.1	1321	valosin containing protein
