# Supplementary material for: Insights into the signal transduction pathways of mouse lung type II cells revealed by transcription factor profiling in the transcriptome
Source: Genomics Inform. 2019 Mar 31;17(1):e8. doi: 10.5808/GI.2019.17.1.e8 (PMC6459171; doi:10.5808/GI.2019.17.1.e8)
Supplement: Supplementary Fig. 1. — Cluster analysis of gene expression in lung type II cells in comparison with other mouse tissues. [file gi-2019-17-1-e8-suppl2.pdf]

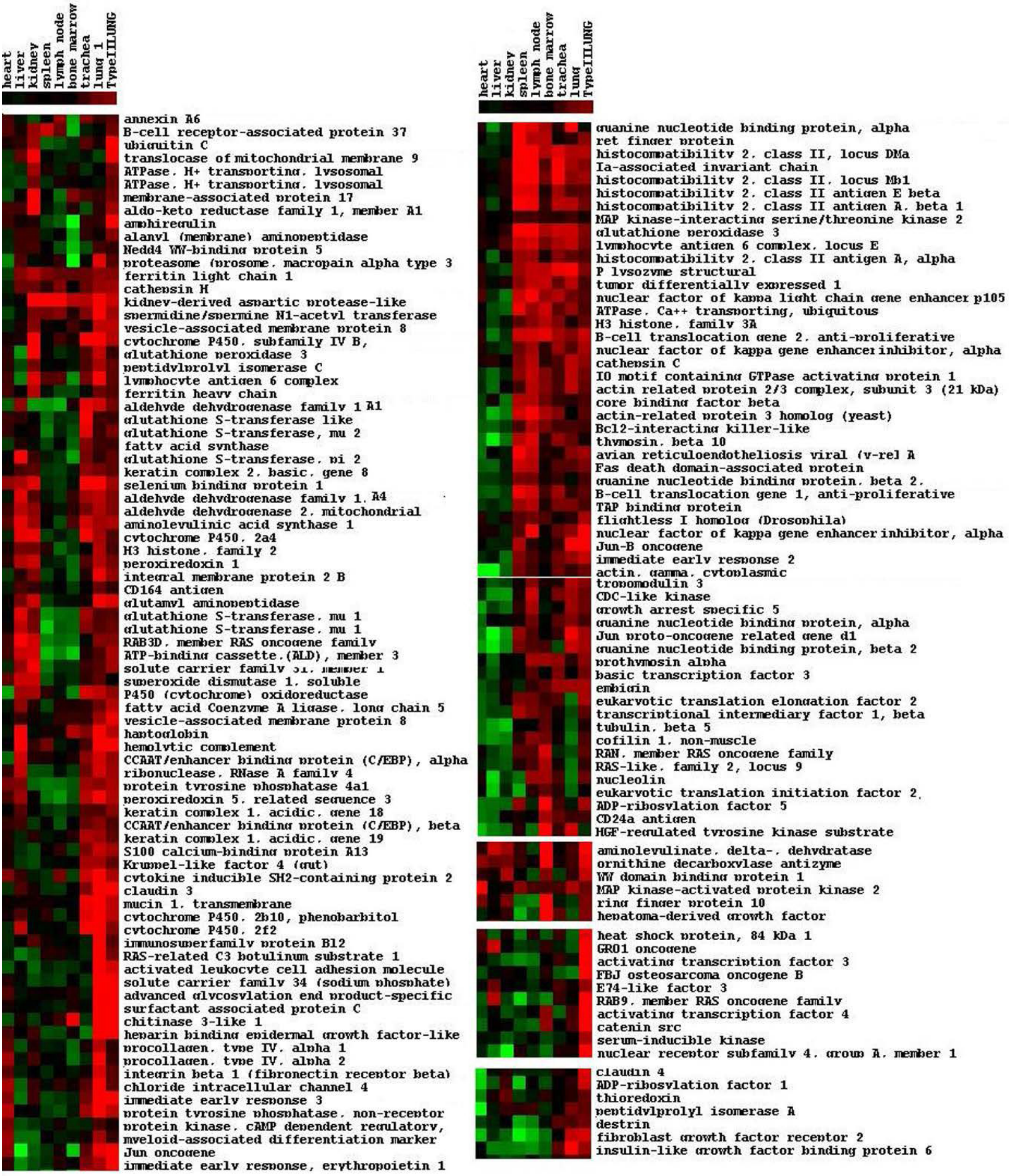

Supplementary Fig. 1. Cluster analysis of gene expression in lung type II cells in comparison with other mouse tissues.
